# Coronary revascularisation in a patient with dextrocardia with situs inversus

**DOI:** 10.1186/1749-8090-8-S1-P35

**Published:** 2013-09-11

**Authors:** MS Siddiqi

**Affiliations:** 1 Department of Cardiothoracic Surgery, Sultan Qaboos University Hospital, Muscat, Oman

## Background

A few cases of CABG surgery in patients with dextrocardia and situs inversus have been reported so far worldwide. This is the first reported case of CABG surgery, in patient with dextrocardia and situs inversus performed in Middle East.

## Methods

The surgery was done with surgeon standing on the left side. Vessels revascularized included the right internal mammary artery to the left anterior descending artery, the saphenous vein to the diagonal artery and to the obtuse marginal artery.

## Results

The patient was discharged to his home after 12 days post operative with no complication. He was doing well after 3 months of follow-up.

## Conclusions

Performing a coronary artery bypass surgery in patient with dextrocardia and situs inverses is a challenging case, in the fact it may be technically more demanding. However, it can be performed with success in same manner CABG is preformed in a non-dextrocardia patient if adequate modifications are made during surgery.

